# Disentangling comorbidity: symptom dimensions of internalizing and functional disorders in a large general population sample

**DOI:** 10.1186/s12888-026-07797-5

**Published:** 2026-01-16

**Authors:** Urvi Saini, Steven H. Aggen, Albertine J. Oldehinkel, Judith G. M. Rosmalen, Hanna M. van Loo

**Affiliations:** 1 Department of Psychiatry, Interdisciplinary Center Psychopathology and Emotion Regulation (ICPE), University of Groningen, University Medical Center Groningen, Hanzeplein 1, PO Box 30001, Groningen, 9700 RB The Netherlands; 2 Virginia Institute for Psychiatric and Behavioral Genetics, Department of Psychiatry, Virginia Commonwealth University, Richmond, VA USA; 3 Department of Internal Medicine, University of Groningen, University Medical Center Groningen, Groningen, The Netherlands

**Keywords:** Depression, Anxiety, Chronic fatigue syndrome, Fibromyalgia, Irritable bowel syndrome, Factor analysis, Symptom dimensions, Comorbidity, Functional somatic disorders

## Abstract

**Background:**

Internalizing disorders (IDs) and functional disorders (FDs) are highly comorbid, however the mechanisms underlying their co-occurrence remain unclear. This study investigated the symptom dimensions of major depressive disorder (MDD), generalized anxiety disorder (GAD), myalgic encephalomyelitis/chronic fatigue syndrome (ME/CFS), fibromyalgia (FM), and irritable bowel syndrome (IBS) symptoms in the general population to understand comorbidity at the symptom level.

**Method:**

We analyzed cross-sectional data from 108,418 adult participants in the Dutch Lifelines Cohort Study, including 30 symptoms from ID and FD diagnostic criteria. The sample was randomly split into two subsets (*n* = 54,209 each), with exploratory factor analysis conducted in the training set and confirmatory factor analysis in the testing set. Associations between the identified symptom dimensions and known risk factors (e.g., demographics, lifestyle, stress) for IDs and FDs, as well as their alignment with ID and FD diagnoses based on diagnostic criteria, were examined.

**Results:**

Five symptom dimensions best captured the structure of ID and FD symptoms: depression, anxiety, IBS, musculoskeletal pain, and general malaise. Symptoms of ME/CFS and FM did not form separate symptom dimensions but loaded on two distinct dimensions: musculoskeletal pain and general malaise. The general malaise dimension mostly captured the transdiagnostic symptoms that were part of almost all ID/FD diagnoses, such as concentration difficulty, fatigue and unrefreshing sleep, plus a few symptoms from the ME/CFS criteria. This general malaise dimension was correlated with every other dimension (excluding the IBS dimension) and associated with all five disorders. Chronic stress was the one risk factor associated with all dimensions.

**Conclusions:**

The diagnostic symptoms of IDs and FDs load on four disorder-specific dimensions and one dimension with transdiagnostic symptoms, general malaise. The general malaise dimension may be central to disorder comorbidity and a key target for further research.

**Clinical trial number:**

Not applicable.

**Supplementary Information:**

The online version contains supplementary material available at 10.1186/s12888-026-07797-5.

## Introduction

Internalizing disorders (IDs) are mental health conditions characterized by emotional, cognitive, and behavioral symptoms that are primarily directed inward rather than outward [1]. The most common IDs, major depressive disorder (MDD) and generalized anxiety disorder (GAD), often co-occur [2]. While shared genetic and environmental risk factors have been suggested to underlie MDD and anxiety disorders [3, 4], distinct features, including differences in prevalence and genetic associations, have also been observed [5, 6].

Functional disorders (FDs) are defined by persistent physical symptoms over several months, which cannot be diagnosed based on known objectifiable pathological abnormalities [7]. Three common, prototypical FDs are myalgic encephalomyelitis/chronic fatigue syndrome (ME/CFS), fibromyalgia (FM), and irritable bowel syndrome (IBS). Their high co-occurrence and substantial diagnostic overlap [8, 9] suggest shared pathogenetic underpinnings. However, disorder-specific risk factors [10] and differences in genetic profiles [11] have also been identified.

### Mechanisms underlying ID and FD comorbidity

High comorbidity rates between IDs and FDs are well established [12, 13]. Individuals with FDs are more likely to have mood and anxiety disorders than those without (ORs = 3.9–12.6) [14]. Moreover, a population-based study found that individuals with FDs not only have a higher prevalence of IDs compared to those with general medical illnesses (e.g., diabetes, rheumatoid arthritis, inflammatory bowel disease) but also experience greater functional disability [15]. Although the mechanisms underlying the co-occurrence of IDs and FDs remain unclear, previous research suggests multiple possible explanations for their co-occurrence. One is that IDs and FDs share liability to disease due to genetic and biological overlap [16–19]. For example, a study using Swedish registry data found that individuals with ME/CFS, FM, or IBS carry greater genetic risk for IDs [11]. Another possibility is that the presence of one disorder triggers the onset of the other [13, 20, 21]. Supporting this, one study demonstrated the causal effects of MDD and IBS on each other [22], while another found FM to predict MDD over time but not vice versa [23].

While previous studies have provided valuable insights into the mechanisms driving ID and FD comorbidity, they have primarily focused on the disorder level or used clinical samples. This approach overlooks disorder heterogeneity, as individuals can meet diagnostic criteria for the same disorder through different combinations of symptoms. It is possible that high comorbidity rates are partly driven by shared symptoms across diagnostic criteria [8]. A symptom-level approach makes it possible to identify patterns and interactions among symptoms that disorder-level analyses might miss. Furthermore, examining ID and FD diagnostic symptoms in the general population avoids the constraints of imposing diagnostic thresholds, allowing us to observe natural symptom (co-)occurrence. Collectively, identifying these symptom-level relationships can provide a deeper understanding of the shared and distinct structural organization contributing to ID and FD comorbidity, as different symptom patterns may reflect distinct underlying pathways [24].

### Symptom level investigation of ID and FD comorbidity

In a previous study, we examined the symptom network connectivity of IDs and FDs in the general population [25]. The network model showed two distinguishable clusters of ID and FD symptoms, linked through their shared symptoms (notably fatigue, trouble sleeping, unrefreshing sleep, and difficulty concentrating). Only a few direct connections between other ID and FD symptoms were observed. Network models capture only direct associations between symptoms, without modeling shared variance of multiple symptoms [26]. In other words, although the ID and FD symptom clusters were not directly connected in the network, their co-occurrence may still be examined as shared variance attributable to one or more latent dimensions that are not explicitly modeled. With latent factor models, it is possible to account for this shared variance and investigate whether ID and FD symptoms reflect separate or overlapping underlying dimensions [27]. Previous studies have demonstrated the multidimensionality of IDs and FDs separately [28–32], however, none have yet examined their structure simultaneously. The joint investigation of ID and FD diagnostic symptoms can reveal underlying dimensional structures that may cut across traditional diagnostic boundaries and provide a foundation for further research into their distinct and shared underlying mechanisms.

### Study aims

The primary aim of this study was to investigate the dimensional structure of the combined diagnostic symptoms of IDs and FDs in the general population. Studying these symptoms in the general population enables the identification of underlying symptom dimensions without the biases typically associated with clinical samples, such as diagnostic bias in physicians (e.g., more diagnoses given to patients who comply with the stereotyped characteristics of the disorder) and help-seeking behavior [33, 34], thus providing a broader picture of how these symptoms and the disorders they define manifest in the general population. The secondary aim was to explore how the identified dimensions relate to established correlates of IDs and FDs (e.g., sex, age, chronic stress), to identify patterns of shared and unique liability that may provide insight into the mechanisms underlying comorbidity [5, 10, 14, 15]. We also examined current diagnostic status of IDs and FDs to evaluate how the empirically derived liability dimensions align with the consensus based diagnostic criteria for these disorders.

## Methods

### Participants

#### Lifelines cohort study

All data were obtained from Lifelines. Lifelines is a multidisciplinary prospective population-based cohort study examining the health and health-related behaviors of 167,729 persons living in the North of the Netherlands in a unique three-generation design [35]. It employs a broad range of investigative procedures in assessing the biomedical, socio-demographic, behavioral, physical and psychological factors that contribute to the health and disease of the general population, with a special focus on multi-morbidity and complex genetics. The detailed description of Lifelines’ recruitment procedure has been described previously [35–37]. The Lifelines cohort study has been approved by the Medical Ethical Committee of the University Medical Center Groningen. All participants provided written informed consent for participation.

#### Participants

We analyzed data collected during the wave two assessment of Lifelines (2014–2019). A total of 108,418 participants aged 18 years and older with available symptom data for IDs and/or FDs were included.

### Measures

#### Internalizing disorders

The Mini International Neuropsychiatric Interview (MINI) was used to assess MDD and GAD symptoms as defined by the fourth edition of the Diagnostic and Statistical Manual of Mental Disorders (DSM-IV) [38]. The twelve MDD symptoms included were depressed mood, anhedonia, appetite change, weight gain, weight loss, trouble sleeping, psychomotor retardation, psychomotor agitation, fatigue, worthlessness/guilt, concentration difficulty, and suicidal ideation. For GAD, the seven symptoms included excessive worry/anxiety which was difficult to control, restlessness, muscle tension, fatigue, concentration difficulty, irritability, and trouble sleeping. All symptoms were reported in a yes/no format with yes being coded as 1 (present) and no as 0 (absent).

#### Functional disorders

ME/CFS symptoms were examined using the 1994 Centers for Disease Control and Prevention (CDC) criteria [39]. To meet the diagnostic criteria, participants had to report chronic fatigue for at least six months, with the fatigue significantly interfering with daily activities and work. In addition, participants had to report at least four of the eight additional symptoms: sore throat, tender lymph nodes, muscle pain, joint pain, headaches, unrefreshing sleep, post-exertional malaise, and impaired memory or impaired concentration. Impaired memory and impaired concentration were combined into a single symptom (‘concentration difficulty’) in accordance with the CDC criteria, which require at least one of the two symptoms to be present.

FM symptoms were examined using the 2010 American College of Rheumatology (ACR) criteria [40]. To meet the diagnostic criteria for FM, participants had to have experienced pain symptoms for at least three months and either have a Widespread Pain Index (WPI) sum score ≥ 7 and symptom severity (SS) score ≥ 5 or a WPI sum score between 3 and 6 and SS score ≥ 9. The WPI measured which of the 19 stated body areas participants had experienced pain during the last week [40]. The SS score was based on the Checklist Individual Strength (CIS) and the somatization scale of the Symptom Checklist-90 (SCL-90 SOM) (Supplemental Methods). The CIS assessed fatigue and cognitive symptoms in the past two weeks [41]. The SCL-90 SOM symptoms are not specific to the FM diagnostic criteria but instead represent a limited selection from the wide range of somatic symptoms an individual may experience. The symptoms from this scale were therefore not included in the analysis of symptom dimensions.

IBS symptoms were assessed using the Rome III IBS Diagnostic Questionnaire [42], with the criteria later adjusted to align with the current ROME IV criteria [43]. To meet the diagnostic criteria for IBS, participants were required to report recurrent abdominal pain or discomfort at least one day per week for at least 6 months, in addition to at least two of the three additional symptoms (pain improvement with defecation, onset of pain/discomfort associated with a change in stool frequency, and onset of pain/discomfort associated with a change in stool appearance). These three additional symptoms were only assessed if recurrent abdominal pain was present. For the analysis, if participants did not have abdominal pain, their conditional symptoms were coded as missing.

All FD symptoms were measured through self-report questionnaires. To ensure comparability with our previous network analysis, which required binary symptom data [25], all FD symptoms were dichotomized in the present study (Supplemental Methods).

### External variables

We further explored associations of the identified symptom liability dimensions with variables covering three domains, selected based on prior evidence demonstrating links with both IDs and FDs: (a) Demographics: sex and age [5, 14, 44–46]; (b): Lifestyle: body mass index (BMI) and heavy drinking [10, 45–51]; and (c) Environmental adversity: chronic stress in the past year (e.g., work, home, relationship stress) [10, 45, 46, 52], acute stress in the past year (e.g., severe injury, death of a loved one, divorce) [10, 45, 46, 53, 54], and childhood sexual abuse [55–58]. Assessment details and definitions can be found in the Supplemental Methods.

In addition, we included current diagnostic status for MDD, GAD, ME/CFS, FM, and IBS as well as other medical conditions. Other medical conditions were included to account for potential confounding by diseases that share symptoms and risk factors with the IDs and FDs, and comprised self-reported presence of cancer, rheumatoid arthritis, osteoarthritis, celiac disease, heart failure, Crohn’s disease, and ulcerative colitis.

### Statistical methods

#### Symptom aggregation

The diagnostic criteria for IDs and FDs have some overlapping symptoms. These transdiagnostic symptoms and the disorders they present in were trouble sleeping (MDD, GAD), unrefreshing sleep (ME/CFS, FM), fatigue (MDD, GAD, ME/CFS, FM), and concentration difficulty (MDD, GAD, ME/CFS, FM). In accordance with our previous paper [25], we combined the overlapping symptoms to avoid multicollinearity leading to unreliable or uninformative factor structures and misinterpretation of the models [59]. If a symptom was reported as present in at least one measure, the combined symptom was coded as 1 (present). In the WPI, the specific body areas affected by pain are not relevant in the context of the diagnostic symptom criteria of FM, but the number of pain areas is. Therefore, the 19 binary WPI items used to define FM were combined into a sum score (ranging from 0 to 19) and dichotomized into 1 if the total score was ≥ 7 and 0 if the total score was < 7, following the FM diagnostic algorithm threshold.

#### Statistical analysis

A common factor modeling approach was used to examine the structure of the individual dichotomously coded ID and FD symptoms [60]. The data was randomly split into two equal subsets, a training set (*n* = 54,209) and a validation set (*n* = 54,209), using function sample() in R version 4.2.1 [61]. An exploratory factor analysis (EFA) was first conducted on the training set to identify the optimal factorial structure. The EFA was performed on all included symptoms. Maximum Likelihood Robust (MLR) method was used to estimate factor loadings and standard errors that are robust to non-normality and non-independence of the observations. Monte Carlo integration was used to randomly generate numerical integration points to obtain maximum likelihood parameter estimates. Geomin oblique rotation method was used to maximize high loadings and minimize low loadings, providing a more interpretable solution [62]. This method allows the factors to correlate and provide a more realistic and interpretable model. For interpretation purposes, correlations and loadings < |0.30| were considered as weak, between |0.30| to |0.40| as moderate, and ≥ |0.40| as strong [63]. The optimal number of factors from the EFA were chosen based on a combination of the fit indices, Kaiser criterion (eigenvalue-greater-than-one rule) [64], and the interpretability of the factors.

**Fig. 1:**
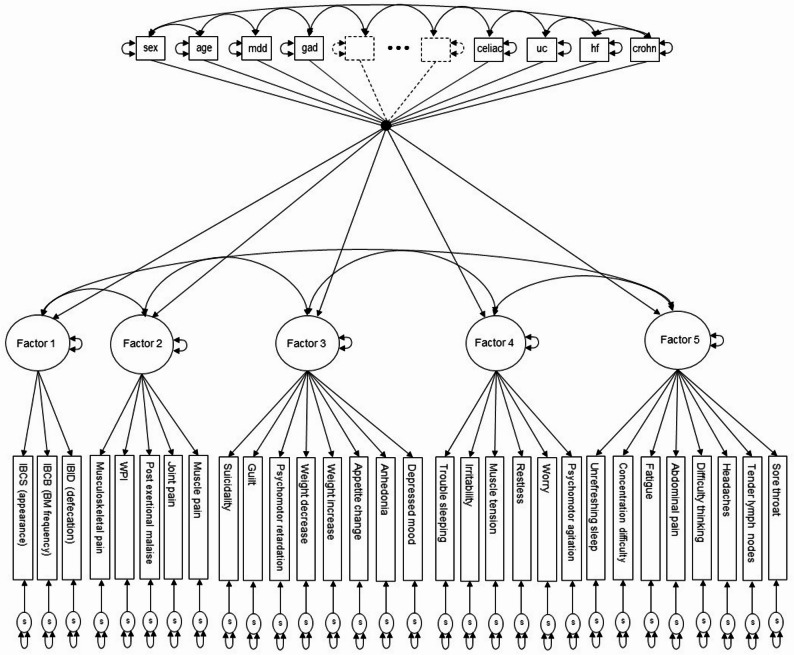
Path diagram of the final five-factor CFA model with covariates. Path diagram of the final five-factor CFA model with covariates. Rectangular and square boxes denote observed binary ID and FD symptom indicator variables and the covariates, respectively. Circles indicate latent variables (factors) and the single-headed arrows from the factors to the rectangular boxes are factor loadings. Single-headed arrows from the square boxes (covariates) to the circles (factors) are partial regression coefficients. Two-headed arrows between different boxes or circles are correlations. The two-headed arrow attached to a box or circle indicates the variance of that variable. Circles at the bottom of the figure with single-headed arrows pointing towards the binary indicator variables are the individual symptom specifics (reliable unique variance plus random measurement error). The two dashed-lined squares with directed arrows and the “***” notation are used to depict covariates that are not explicitly shown. The single dot “•” into which and from which covariate regression lines enter and leave is used to reduce graphic clutter that would result when drawing all the directed arrows from each covariate to each factor.

After having identified an optimal and interpretable factorial structure from the EFA analysis, we conducted a confirmatory factor analysis (CFA). To preserve full symptom coverage across diagnostic categories, we did not set a minimum loading cut-off, meaning that each symptom was retained in the CFA. Each symptom was constrained to load only on the factor for which it showed the highest absolute factor loading in the EFA. Cross-loadings were restricted to zero, and factor covariances were freely estimated to examine relationships between the latent constructs. The model was first estimated without the covariates to examine the restricted validity of the overall model. Then, the CFA model was estimated while simultaneously regressing the factors onto the covariates to estimate the relationships between the covariates and the latent factors (Fig. 1). Covariate effect sizes are reported as standardized partial regression coefficients (*β*) and interpreted as the estimated number of standard deviations of change in the dependent variable (symptom dimension score) for one standard deviation unit change in the independent variable (covariate) [65]. For binary covariates, *β* is interpreted as the change in the dependent variable when the binary covariate changes from 0 to 1.

#### Factor interpretation

The general population sample in this study includes both individuals whose self-reported symptoms meet algorithmic diagnostic criteria and those who do not. Therefore, the identified latent factors should be interpreted as liabilities for specific symptom patterns rather than indicators of dimensional severity. This framing conceptualizes the factors as underlying symptom-defined liabilities that may inform, but are not equivalent to, diagnostic classifications.

All analyses were performed in MPlus version 8.2 [65].

#### Handling of missing data

In the full data set (*n* = 108,418), the three IBS conditional symptoms (pain improvement with defecation, onset of pain/discomfort associated with a change in stool frequency, and onset of pain/discomfort associated with a change in stool appearance) had the greatest number of missing values (61.7%); for all other symptoms the percentage ranged from 15.5% to 25.0% (Supplemental Table 1). The large proportion of missing data for the conditional IBS symptoms was due to a skip out rule used in the questionnaire: if participants did not have abdominal pain, they did not answer the other three IBS symptoms. These conditional symptoms were therefore coded as missing in that case.

To address missing data on ID and FD symptoms, we used full information maximum likelihood (FIML), implemented using a Monte Carlo integration approach, for the EFA and CFA. This estimation approach uses all available data to produce unbiased parameter estimates, therefore improving statistical power and ensuring consistency in the estimation of factor loadings and model fit indices [66]. Simultaneous FIML to jointly handle missingness for both symptom and covariate data was deemed not feasible due to the high computational demands of numerical integration. Therefore, missing values on covariates were imputed using multiple imputation in Mplus [65]. Ten imputed data sets were generated using the sequential regression method (i.e., multivariate imputation by chained equations) [67]. The CFA with covariates was then estimated in each imputed data set using FIML, with parameter estimates pooled and the standard errors calculated according to Rubin’s rule [68].

## Results

### Sample description

In the full sample (*n* = 108,418), 58.6% of the participants were female and the average age was 49.3 years (SD = 13.0) (Table 1). 13.7% of participants met the diagnostic criteria for at least one disorder. Point prevalence was highest for FM and GAD, and lowest for MDD and ME/CFS. The overlapping symptoms were the most common in the sample, with 47.3% reporting fatigue, 39.9% concentration difficulty, 38.0% unrefreshing sleep, and 31.8% trouble sleeping (Supplemental Table 1). The least prevalent symptoms were the MDD criteria psychomotor retardation and suicidality, both reported by 1.1%.

**Table 1:** Sample description (*n* = 108,418)

Variable	Mean (SD)	Min	Max	% Missing
*N* = 108,418				
**Demographics**				
Age	49.3 (13.0)	18.0	96.0	0.0
Sex (% female)	58.6	---	---	0.0
Diagnostic status (%)*				
MDD	3.0	---	---	15.5
GAD	6.0	---	---	15.5
ME/CFS	3.1	---	---	17.8
FM	6.6	---	---	24.9
IBS	5.5	---	---	17.5
**Risk factors**				
BMI	26.0 (4.1)	12.9	43.3	0.4
Chronic stress	1.8 (1.8)	0.0	12.0	10.1
Acute stress	0.8 (1.1)	0.0	12.0	12.1
Heavy drinking (%)	4.7	---	---	17.2
Childhood sexual abuse (%)	7.1	---	---	42.4
**Other medical conditions (%)**				
Cancer	5.8	---	---	0.4
Rheumatoid arthritis	2.6	---	---	1.8
Osteoarthritis	12.0	---	---	1.8
Celiac disease	0.5	---	---	6.8
Heart failure	1.3	---	---	2.8
Crohn’s disease	0.4	---	---	3.1
Ulcerative colitis	0.7	---	---	3.1

Means are given for continuous variables and percentages for binary variables. SD = standard deviation. Minimum and maximum values are provided for continuous variables. % Missing = percentage of missing data for the variables. Heavy drinking is defined as ≥ 4 drinks per day for women and ≥ 6 drinks per day for men at least once a week in the past month (Supplemental materials). *Point prevalence estimates reflect disorder status at a specific assessment time point rather than across the whole lifespan and are usually lower than lifetime prevalences, especially for disorders with an episodic nature, such as MDD and GAD.

### ID and FD symptom structures

First, EFAs of the 30 symptoms were conducted in the training data set (*n* = 54,209). Due to the power available with large sample sizes, EFA fit indices consistently improved with additional factors being extracted (Table 2). However, solutions with six or more factors yielded eigenvalues below one (Supplemental Fig. 1), and were difficult to interpret (e.g., the seven-factor model contained a factor defined by a single item). Solutions with six or more factors were therefore not considered. Based on the Kaiser criterion and interpretability of the model, the 5-factor model was selected for further analysis.

**Table 2:** EFA and CFA model fitting results

Model	*N* par	Log LL	AIC	BIC	sBIC
EFA models (*n* = 54,209)					
EFA 1	60	-366852.9	733825.7	734359.8	734169.1
EFA 2	89	-355885.7	711949.4	712741.6	712458.7
EFA 3	117	-348730.5	697695.1	698736.4	698364.6
EFA 4	144	-347279.3	694846.6	696128.3	695670.7
** EFA 5**	**170**	**-345320.6**	**693981.2**	**692494.3**	**691954.0**
EFA 6	195	-344520.8	689431.6	691167.2	690547.5
EFA 7	219	-344069.0	688576.0	690525.2	689829.2
CFA models (*n* = 54,209)					
CFA 5	70	-349173.1	698486.2	699109.3	698886.8
CFA5 + covariates	165	-327538.5	655407.0	656875.6	656351.2

Sample was split equally and the EFA was performed in the training set and the CFA in the testing set. N par = number of parameters; Log LL = raw data log likelihood; AIC = Akaike information criteria; BIC = Bayesian information criterion; sBIC = sample adjusted BIC. All models were estimated using MLR with 4,000 Monte Carlo numerical integration points

In the EFA, all symptoms except IBS abdominal pain loaded > 0.30 on at least one factor (Table 3). Factor 1, representing IBS symptoms, showed strong loadings for the three conditional IBS items (range: 0.80–0.91), but not for the core symptom, abdominal pain. Factor 2 reflected musculoskeletal pain symptoms, with high loadings for pain items from the FM and ME/CFS criteria and for post exertional malaise from the ME/CFS criteria (range: 0.66–0.88). Factor 3, representing depression symptoms, included nearly all non-overlapping MDD items (range: 0.47–0.88) except psychomotor agitation. Factor 4, capturing anxiety symptoms, comprised all four non-overlapping GAD items (range: 0.53–0.99) as well as the MDD item psychomotor agitation (0.53). Trouble sleeping, an overlapping symptom of MDD and GAD, loaded higher on this factor (0.36) than on any of the other factors. Factor 5 encompassed the transdiagnostic symptoms unrefreshing sleep (0.71), fatigue (0.66), and concentration difficulty (0.38). Additionally, difficulty thinking from the FM criteria (0.55), sore throat (0.45), tender lymph nodes (0.47), and headaches (0.42) from the ME/CFS criteria, and abdominal pain from the IBS criteria (0.29) also loaded on this factor. These symptoms reflect more commonly experienced general states of physical and mental disturbances; therefore, we labeled this factor a general malaise factor.

The strongest correlating factors were depression and anxiety (*r* = 0.68), followed by the general malaise factor with the anxiety (*r* = 0.60), musculoskeletal pain (*r* = 0.52), and depression (*r* = 0.48) factors. The musculoskeletal pain factor was moderately correlated with both the depression (*r* = 0.33) and anxiety (*r* = 0.31) factors. Notably, the IBS symptom factor was weakly correlated with the other factors.

Next, we conducted a CFA to further investigate the 5-factor model (Fig. 1; Table 2). The patterning of factor loadings and inter-factor correlations were reasonably consistent with the EFA results, supporting the stability of the 5-factor structure (Table 3). All items had standardized loadings > 0.40 on their respective factors. Allowing no cross-loadings for symptoms in the CFA resulted in higher inter-factor correlation estimates for all factors (0.40 < *r* < 0.82), as expected [60]. The larger fit indices in the CFA compared to the EFA reflect a more restrictive structure with fewer estimated parameters in the CFA. The IBS factor correlated minimally with the other factors.

**Table 3:** 5-factor EFA and CFA loadings

Item	Exploratory factor analysis					Confirmatory factor analysis				
	IBS SF	Pain SF	Dep SF	Anx SF	GM SF	IBS SF	Pain SF	Dep SF	Anx SF	GM SF
**MDD symptoms**										
Depressed mood	0.001	0.056*	0.824*	0.178*	-0.083*	---	---	0.932*	---	---
Anhedonia	-0.015	0.027*	0.875*	0.089*	-0.015	---	---	0.917*	---	---
Appetite change	0.035*	-0.062*	0.635*	0.045	0.170*	---	---	0.720*	---	---
Weight increase	0.045*	0.084*	0.465*	-0.052*	0.084*	---	---	0.476*	---	---
Weight decrease	0.069*	-0.032	0.526*	0.015	0.052	---	---	0.541*	---	---
Psychomotor retardation	-0.004	0.124*	0.557*	-0.093*	0.406*	---	---	0.786*	---	---
Psychomotor agitation	-0.001	0.030*	0.242*	0.533*	0.102*	---	---	---	0.816*	---
Guilt	-0.019	-0.005	0.592*	0.336*	0.044*	---	---	0.877*	---	---
Suicidality	-0.015	-0.008	0.637*	0.290*	-0.027	---	---	0.828*	---	---
**GAD symptoms**										
Worry	-0.018	-0.024*	0.228*	0.630*	0.132*	---	---	---	0.883*	---
Restless	0.012	0.020*	0.028*	0.926*	-0.006	---	---	---	0.944*	---
Muscle tension	0.002	0.030*	-0.050*	0.997*	-0.028*	---	---	---	0.922*	---
Irritability	0.025*	-0.028*	0.168*	0.564*	0.180*	---	---	---	0.806*	---
**ME/CFS symptoms**										
Sore throat	0.013	0.179*	-0.110*	-0.005	0.446*	---	---	---	---	0.501*
Tender lymph nodes	0.012	0.298*	-0.155*	0.052	0.469*	---	---	---	---	0.593*
Muscle pain	-0.001	0.779*	-0.026	0.040*	0.037*	---	0.801*	---	---	---
Joint pain	-0.015	0.875*	0.018	0.010	-0.091*	---	0.799*	---	---	---
Headaches	0.003	0.210*	0.031	0.031	0.418*	---	---	---	---	0.572*
Post exertional malaise	-0.013	0.766*	0.121*	-0.059*	0.132*	---	0.867*	---	---	---
**FM symptoms**										
WPI	0.047*	0.659*	-0.030	0.071*	0.161*	---	0.779*	---	---	---
Difficulty thinking	-0.006	-0.077*	0.263*	0.002	0.554*	---	---	---	---	0.661*
Musculoskeletal pain	-0.008	0.754*	0.093*	-0.014	-0.032*	---	0.769*	---	---	---
**IBS symptoms**										
Abdominal pain	0.110*	0.214*	-0.013	0.084*	0.294*	---	---	---	---	0.481*
IBID (defecation)	0.797*	-0.024*	-0.030	0.030	-0.044*	0.770*	---	---	---	---
IBCB (BM frequency)	0.906*	0.012	0.012	-0.027*	0.024*	0.910*	---	---	---	---
IBCS (appearance)	0.912*	0.010	0.021	-0.004	0.002	0.917*	---	---	---	---
**Overlapping symptoms**										
Trouble sleeping	0.015	0.095*	0.084*	0.357*	0.258*	---	---	---	0.652*	---
Fatigue	-0.022*	0.021*	0.239*	0.095*	0.658*	---	---	---	---	0.875*
Concentration difficulty	0.025*	-0.002	0.323*	0.152*	0.378*	---	---	---	---	0.728*
Unrefreshing sleep	-0.024*	0.018	0.028	0.022	0.713*	---	---	---	---	0.685*
**Factor**	**F1**	**F2**	**F3**	**F4**	**F5**	**F1**	**F2**	**F3**	**F4**	**F5**
1	1.000					1.000				
2	0.133*	1.000				0.166*	1.000			
3	0.054*	0.330*	1.000			0.136*	0.412*	1.000		
4	0.121*	0.307*	0.680*	1.000		0.162*	0.417*	0.820*	1.000	
5	0.145*	0.524*	0.482*	0.602*	1.000	0.175*	0.600*	0.779*	0.821*	1.000

IBS SF = IBS symptom factor; Pain SF = musculoskeletal pain symptom factor; Dep SF = depression symptom factor; Anx SF = anxiety symptom factor; GM SF = general malaise symptom factor. Bolded values indicate the highest absolute factor loading of that symptom across all factors. Factor inter-correlations are presented at the bottom of the table. *Factor loading estimate statistically significant at the p-value < 0.05 level

**Fig. 2:**
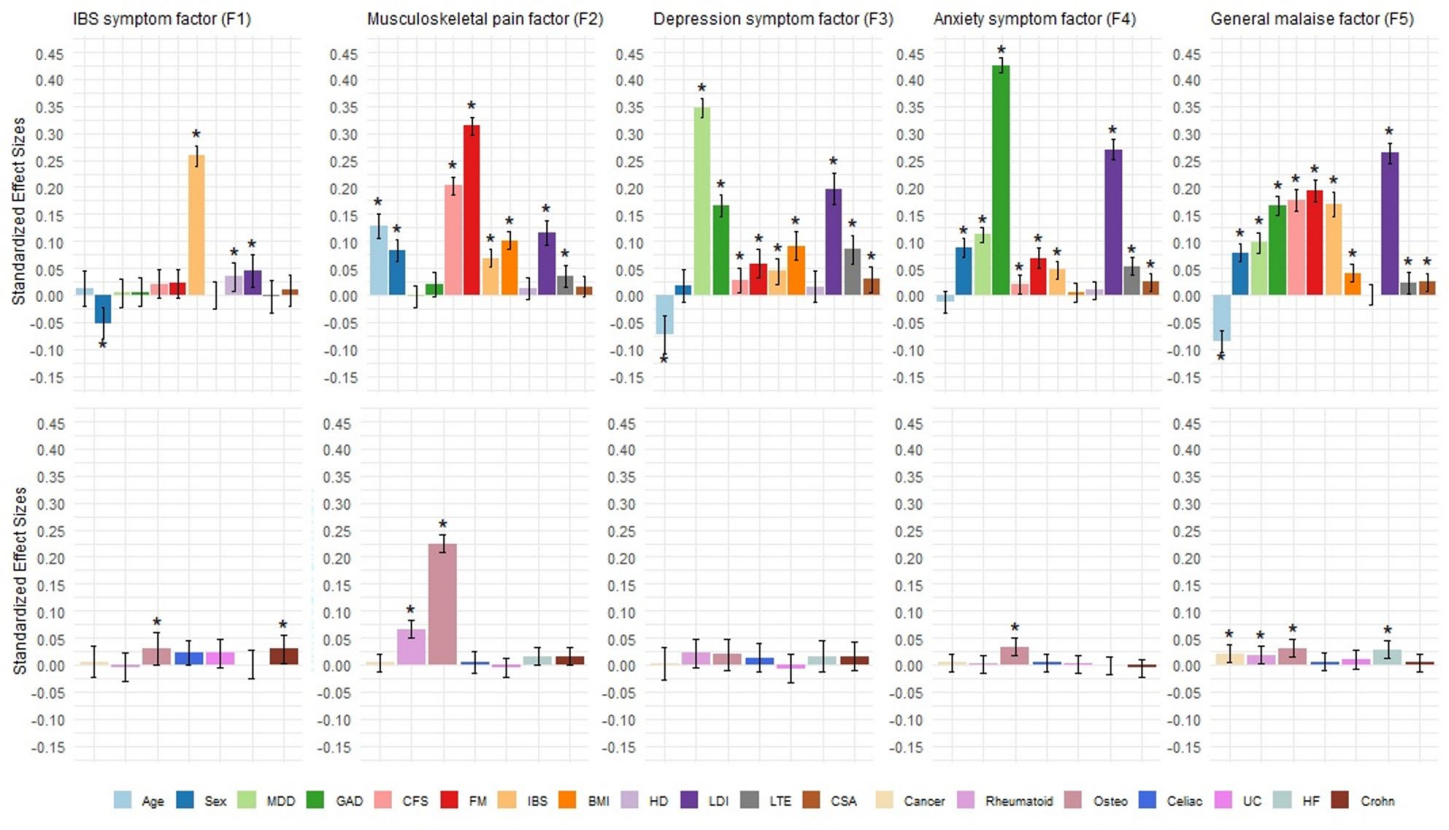
Summary of CFA model standardized partial regression effect sizes for covariates. Summary of CFA model standardized partial regression effect sizes for covariates predicting the five symptom factors. Top row are effect sizes for risk factors and current diagnosis of IDs/FDs. Bottom row are effect sizes for self-reported presence of other medical conditions. Osteo = osteoarthritis; Celiac = Celiac disease; UC = ulcerative colitis; HF = heart failure; Crohn = Crohn’s disease. β coefficients for age are presented per 10 years. *Effect size estimates significant at the p-value < 0.001 level

Adding the external variables in the CFA accounted for additional variance, thereby improving model fit. The unstandardized and standardized effect sizes for the risk factors and ID/FD diagnosis were estimated while adjusting for the influence of other medical conditions (Fig. 2; Supplemental Table 2). The IBS factor associated most strongly with IBS diagnosis (β = 0.26) and weakly with all other external variables (β < 0.03). The musculoskeletal pain factor was primarily associated with FM diagnosis (β = 0.31), followed by osteoarthritis (β = 0.22), ME/CFS diagnosis (β = 0.20), and older age (β = 0.13). The depression factor showed its strongest association with MDD diagnosis (β = 0.35) and the anxiety factor with GAD diagnosis (β = 0.43). Both factors displayed similar associations with the risk factors. However, compared to the anxiety factor, the depression factor had stronger associations with being younger (β=-0.07), BMI (β = 0.09), and acute stress (β = 0.09), whereas the anxiety factor showed stronger associations with being female (β = 0.09) and chronic stress (β = 0.27). Similar to the anxiety symptom factor, the general malaise factor associated strongly with chronic stress (β = 0.26). Notably, all ID and FD diagnoses were relatively strongly associated with this factor (β > 0.10), with the largest association being for FM (β = 0.19) and smallest for MDD (β = 0.10).

### Post-hoc sensitivity analysis

A previous study on predictors of FD onset found that illness related chronic stress was significantly associated with ME/CFS, FM, and IBS, while non-illness related chronic stress was not [10]. To evaluate whether the association between chronic stress and the five latent symptom factors was driven by illness related stress items, we performed a sensitivity analysis in which those items were removed. Results were comparable to the original model, with chronic stress remaining significantly associated with all five factors (Supplemental Fig. 2; Supplemental Table 3).

## Discussion

### Summary of main findings

This study investigated the latent structure of ID and FD symptoms in a large general population sample. We identified a five-dimensional liability structure that captured the transdiagnostic and disorder-specific symptoms across IDs and FDs. Three dimensions were largely defined by disorder-specific symptoms (IBS, MDD, GAD) and hence captured symptom liabilities that aligned with established ID and FD diagnoses. This was further highlighted by the relatively strong associations found between each dimension and its corresponding diagnosis (e.g., MDD diagnosis strongly associated with the depression symptom dimension). ME/CFS and FM symptoms did not form distinct dimensions of their own but were mostly captured by a musculoskeletal pain and a general malaise dimension. This latter dimension comprised transdiagnostic symptoms as well as some disorder-specific symptoms, mainly from the ME/CFS criteria but also the IBS core symptom abdominal pain. The dimensions varied in their associations with the risk factors, with chronic stress being the one risk factor associated with all symptom dimensions. The general malaise dimension presented the greatest number of significant associations, and the IBS dimension the fewest.

### Strengths and limitations

To our knowledge, this is the first study investigating symptom dimensions of major IDs and FDs together in a large general population sample. The large sample size allowed us to split the data into two independent subsamples to explore and evaluate the underlying symptom structures in the exploratory data set and validate the model in the confirmatory data set. The consistency of the patterning of factor loadings and correlations between the exploratory and confirmatory models provided evidence that the identified symptom dimensions were stable and generalizable across the training and test data sets.

Several limitations are noteworthy. First, Lifelines consists of data collected on participants living in the north of the Netherlands, who are predominantly native Dutch. This may limit the generalizability of our findings to populations with different cultural and ethnic backgrounds [69]. Second, the three conditional IBS items were only assessed in participants reporting abdominal pain, resulting in a large number of missing data for these symptoms. Although FIML is able to account for missing data patterns, the large degree of missingness in these items may introduce some level of uncertainty in their parameter estimates. Third, the dichotomization of ME/CFS, FM, and IBS symptoms may reduce the sensitivity to more subtle distinctions between symptom patterns, potentially influencing the factor structure. However, given the large sample size in our study, the overall influence of dichotomization is likely modest. Fourth, the symptoms were all assessed during wave 2, but across different time windows (e.g., in the past 2 weeks for MDD, in the past 6 months for GAD, ME/CFS, and IBS, and in the past 3 months for FM), which may have caused systematic differences in the frequency and correlation patterns of the reported symptoms. Additionally, some symptoms may have been experienced simultaneously, whereas others in non-overlapping time frames, which may have affected our factor structure. Nonetheless, symptoms from different diagnoses did not exclusively form their own disorder-specific dimensions (e.g., the anxiety dimension comprised MDD and GAD symptoms), which suggests that the dimensions were not completely determined by differences in time windows. Lastly, the symptom dimensions and their associations with the external variables were estimated using cross-sectional data and therefore directionality cannot be inferred.

### Comparison with previous studies

The current study extends our previous network analysis study [25] by modeling the shared variance among symptoms using a dimensional approach. Consistent with patterns in the symptom network, MDD and GAD symptoms largely formed separate but substantially correlated dimensions, and FD symptoms split into distinct dimensions representing IBS symptoms and a combined ME/CFS and FM pain dimension. The transdiagnostic symptoms (e.g., fatigue, concentration difficulty, and unrefreshing sleep) identified in the network linking the two ID and FD symptom clusters were captured by a general malaise dimension, which was strongly correlated with every other symptom dimension except IBS. Interestingly, whereas the ME/CFS symptoms tender lymph nodes, sore throat and headache, and the IBS symptom abdominal pain were mostly isolated in the network, in the latent factor model, they clustered together with the transdiagnostic symptoms in the general malaise dimension. As factor models reveal symptom groupings based on their shared variance, this suggests that these ME/CFS and IBS symptoms, similar to the transdiagnostic symptoms, reflect co-occurring features in the general population, which limits their specificity as indicators of different IDs and FDs. For example, a population-based study found sore throat to be equally prevalent in individuals meeting and not meeting ME/CFS criteria [8].

While the network model hardly showed any direct connections between ID and FD symptoms, the latent model uncovered meaningful correlations between their underlying dimensions. The largest association was observed between the depression and anxiety dimension, with more modest associations between the ID and FM and ME/CFS dimension. Both models highlight the distinctness of IBS, which formed a separate symptom cluster in the network model and a weakly correlated latent dimension in the factor model. This result may have been impacted by the presence of systematic missing data for the conditional IBS items, which were only asked when participants endorsed the core IBS symptom, abdominal pain.

### ID and FD comorbidity

Our results suggest that the general malaise dimension may play an important role in shaping comorbidity patterns. This dimension was strongly correlated with the depression, anxiety, and musculoskeletal pain dimensions; it may therefore account for much of the overlap typically observed at the diagnostic level, thereby reducing correlations between individual disorder-specific dimensions. Participants with an ID or FD diagnosis had a significantly higher probability of endorsing symptoms defining the general malaise dimension, reflecting an increased burden of these symptoms across the diagnostic groups. This pattern is consistent with findings from Thomas et al. [70], who found that individuals with MDD reporting fatigue, a high loading symptom in the general malaise dimension, had a significant increase in risk of FDs. In addition, although the general malaise dimension was weakly correlated with the IBS symptom dimension, it was significantly associated with IBS diagnosis. This is likely due to this dimension including both abdominal pain, a core symptom required for IBS diagnosis, and the non-specific symptoms that are commonly reported by individuals with IBS (e.g., headaches, fatigue, sleeping disturbances) [71]. The consistent association of the general malaise dimension with both IDs and FDs in the present study suggests that it functions as a transdiagnostic symptom dimension linked to multiple disorders. Future studies using longitudinal data are necessary to determine potential contributions the general dimension may have on shaping comorbidity patterns and reflecting shared pathways.

Our findings also provide evidence for ID and FD potentially sharing other mechanisms that contribute to their frequent co-occurrence. Chronic stress was associated with higher scores on all five dimensions, possibly reflecting dysregulation in the stress response system (i.e., autonomic nervous system and HPA), which is known to underlie both IDs and FDs [72, 73]. Additionally, the close relationship between IDs and ME/CFS is demonstrated by six out of the nine ME/CFS diagnostic symptoms loading on the general malaise dimension, which shares symptoms commonly seen in MDD and GAD. This aligns with previous genetic studies showing that, among FDs, ME/CFS is most closely related to IDs [19]. Notably, the IBS symptom dimension showed no association with other ID and FD diagnoses and only had one risk factor (e.g., chronic stress) in common with the other dimensions, suggesting a relatively distinct pattern in this study.

### Implications and future research

The results of this study have important implications for the classification of IDs and FDs. While the depression and anxiety symptom dimensions mostly captured symptoms belonging to MDD and GAD, respectively, disorder-specific symptom dimensions were lacking for ME/CFS and FM symptoms. Instead, symptoms of these FDs shared the musculoskeletal pain and general malaise dimensions, a finding consistent with ME/CFS and FM being characterized by more similarities than differences [11, 74]. Unsurprisingly, the musculoskeletal pain dimension presented a stronger association with FM than with ME/CFS, reflecting pain as the hallmark symptom of FM. However, the general malaise dimension also showed a stronger association with FM than with ME/CFS, despite it encompassing more ME/CFS symptoms, including its core symptom fatigue. These results reflect findings from previous research showing that fatigue and pain-related ME/CFS symptoms occur frequently in individuals with FM [75], while sore throat and tender lymph nodes are relatively uncommon in those with ME/CFS in population-based samples [76]. Notably, post exertional malaise, often interpreted as the hallmark symptom of ME/CFS, loaded on the musculoskeletal pain dimension and not on the general malaise dimension. Together, these findings suggest that the current symptom-based distinctions between ME/CFS and FM may be less clear-cut than current diagnostic criteria suggest.

The results of this study further our understanding of how symptoms of ME/CFS and FM overlap, but cannot, on their own, determine diagnostic boundaries. The use of arbitrary cut-offs to define the presence or absence of these disorders does not capture their continuous and multidimensional nature. In addition, individuals with risk factors shared across dimensions may present with similar symptoms, further complicating differential diagnosis. Our results indicate that symptom-based criteria alone may not fully differentiate ME/CFS and FM. To better inform their classification decisions, future research integrating longitudinal (e.g., course of illness), etiological (e.g., shared genetics, environmental factors), and treatment response data is necessary [77]. Combined with the empirically derived symptom dimensions identified in this study, these insights could help clinicians and researchers identify the most informative symptoms for diagnosis and further investigation.

Comorbid conditions have consistently been associated with substantial reductions in quality of life [78], stressing the need to better understand these overlaps. Given the significant associations between the general malaise dimension and ID/FD diagnoses, it is tempting to speculate that risk factors influence comorbidity partly through the general malaise dimension. However, to determine directional relationships (risk factors ➜ general malaise dimension ➜ comorbid ID and FD), further longitudinal research is necessary. Future studies should also aim to uncover the genetic architecture underlying these empirically based latent dimensions to identify both shared and unique contributions to IDs and FDs. In addition, examining the symptom dimensions of other FDs that co-occur with IDs (e.g., functional neurological disorder) may further inform our understanding of their shared and distinct underlying mechanisms. Lastly, examining a broader range of risk factors beyond those included here may provide deeper insight into the shared and distinct etiologies underlying these symptom dimensions.

## Conclusion

This study extends current understanding of the multidimensional and continuous nature of IDs and FDs by identifying empirically derived symptom dimensions that cut across traditional diagnostic boundaries. We found three dimensions capturing disorder-specific symptoms of MDD, GAD, and IBS, while ME/CFS and FM symptoms were mostly combined in a musculoskeletal pain and a general malaise dimension. In addition to some ME/CFS symptoms, the general malaise dimension included most transdiagnostic symptoms and IBS abdominal pain, and was associated with all IDs and FDs, suggesting that these symptoms have limited specificity as indicators of different IDs and FDs in a general population. This dimension was also associated with all other symptom dimensions except the IBS dimension, suggesting it may function as a transdiagnostic symptom dimension linking multiple disorders. As such, it may serve as a target for future longitudinal investigations into the mechanisms underlying ID and FD comorbidity.

## Supplementary Information

Below is the link to the electronic supplementary material.

Supplementary Material 1

## Data Availability

The data analyzed in this study may be obtained from a third party and are not publicly available. Researchers can apply to use the Lifelines data used in this study. More information about to request Lifelines data and the conditions of use can be found on their website (https://www.lifelines-biobank.com/researchers/working-with-us).

## Abbreviations

ID: Internalizing disorder
FD: Functional disorder
MDD: Major depressive disorder
GAD: Generalized anxiety disorder
ME/CFS: Myalgic encephalomyelitis/chronic fatigue syndrome
FM: Fibromyalgia
IBS: Irritable bowel syndrome
MINI: Mini International Neuropsychiatric Interview
DSM: Diagnostic and Statistical Manual of Mental Disorders
CDC: Center for Disease Control and Prevention
ACR: American College of Rheumatology
WPI: Widespread Pain Index
SS: Symptom severity
CIS: Checklist Individual Strength
SCL-90 SOM: Somatization scale of the Symptom Checklist-90
BMI: Body mass index
EFA: Exploratory factor analysis
MLR: Maximum Likelihood Robust estimator
CFA: Confirmatory factor analysis
β: Standardized partial regression coefficient
FIML: Full information maximum likelihood
SD: Standard deviation
