# Supplementary material for: Disentangling comorbidity: symptom dimensions of internalizing and functional disorders in a large general population sample
Source: BMC Psychiatry. 2026 Jan 16;26:240. doi: 10.1186/s12888-026-07797-5 (PMC12998144; doi:10.1186/s12888-026-07797-5)
Supplement: Supplementary file 1 — Supplementary Material 1 [file 12888_2026_7797_MOESM1_ESM.docx]

**Supplementary materials**

Table of Contents

**Supplemental methods2**

Symptom dichotomization2

External variables3

**Supplemental results4**

Supplemental Table 14

Supplemental Figure 15

Supplemental Table 2 6

Supplemental Figure 2 7

Supplemental Table 3 8

**References** **9**

**Supplemental Methods**

***Symptom dichotomization***

*ME/CFS symptoms*

Participants rated the symptoms on a scale of 1 to 4 (1 = “not at all”; 4 = “every day”). To dichotomize data, for all items, a score of 1 and 2 were coded as 0 (absent) and scores of 3 and 4 were coded as 1 (present). In Lifelines, impaired memory and impaired concentration were assessed by separate items, which were combined into a single symptom following the CDC criteria that require the presence of either impaired memory or concentration or both. If either impaired memory or impaired concentration was 1, then the combined symptom was 1 and if both were 0, then the combined symptom was 0. If either impaired memory or concentration were NA, then the combined symptom was NA if the other was NA or 0, and 1 if the other was 1.

*FM symptoms*

Checklist Individual Strength (CIS): on a scale of 1 (“yes, true”) to 7 (“no, not true”), participants rated their experiences, indicating the extent to which they generally felt tired, had difficulty thinking, concentration difficulty, and did not wake up feeling rested. To dichotomize the data, a score between 1 to 3 was coded as 1 and a score between 4 to 7 was coded as 0.

Symptom Checklist-90 (SCL-90 SOM): consisted of 12 somatic symptoms, with participants reporting to what extent they were affected by each symptom on a scale of 1 (“not at all”) to 5 (“extremely”).

***External variables***

*Demographics*

**Sex**: binary (0 = Males; 1 = Females).

**Age**: age at assessment (range: 18-96 years).

*Lifestyle*

**Body Mass Index (BMI):** based on self-reported height and weight during wave 2 assessment.

Future research should explore other alternatives, such as the waist-to-hip ratio.

**Heavy drinking:** defined as ≥ 4 drinks per day for women and ≥ 6 drinks per day for men at least once a week in the past month (Statistics Netherlands, 2024).

*Environmental adversity*

**Chronic stress**: measured using the Long-term Difficulties Inventory (LDI) (Rosmalen et al., 2012). The LDI assessed 12 chronic stressors in the past year via self-report.

**Acute stress**: measured using the List of Threatening Experiences (LTE)(Brugha & Cragg, 1990; Rosmalen et al., 2012). The LTE assessed 12 major categories of stressful life events in the past year via self-report.

**Childhood sexual abuse (CSA)**: was assessed with the Childhood Trauma Questionnaire Short Form (CTQ)(Thombs et al., 2009).

**Supplemental Results**

**Supplemental Table 1.** ID and FD symptom frequencies

| Symptom | % | % Miss | Symptom | % | % Miss |
| --- | --- | --- | --- | --- | --- |
| **MDD** |  |  | **FM** |  |  |
| Depressed mood | 4.1 | 15.5 | WPI | 8.6 | 17.3 |
| Anhedonia | 5.5 | 15.5 | Difficulty thinking | 14.6 | 17.6 |
| Appetite change | 4.3 | 15.5 | Musculoskeletal pain | 15.8 | 17.4 |
| Weight gain | 4.7 | 15.5 | **IBS** |  |  |
| Weight loss | 2.3 | 15.5 | Abdominal pain | 11.1 | 17.4 |
| Psychomotor retardation | 1.1 | 15.5 | IBID (defecation)* | 74.6 | 61.6 |
| Psychomotor agitation | 4.7 | 15.5 | IBCB (BM frequency)* | 55.7 | 61.7 |
| Guilt | 3.2 | 15.5 | IBCS (appearance)* | 65.7 | 61.7 |
| Suicidal | 1.1 | 15.5 | **Overlapping Symptoms** |  |  |
| **GAD** |  |  | Trouble sleeping | 31.8 | 15.5 |
| Worry | 6.7 | 15.5 | Fatigue | 47.3 | 22.6 |
| Restless | 16.2 | 15.5 | Concentration difficulty | 39.9 | 25.0 |
| Muscle tension | 25.2 | 15.5 | Unrefreshing sleep | 38.0 | 17.7 |
| Irritability | 18.1 | 15.5 |  |  |  |
| **ME/CFS** |  |  |  |  |  |
| Sore throat | 1.6 | 17.8 |  |  |  |
| Tender lymph nodes | 1.4 | 17.8 |  |  |  |
| Muscle pain | 14.9 | 17.9 |  |  |  |
| Joint pain | 22.0 | 17.9 |  |  |  |
| Headaches | 7.5 | 17.7 |  |  |  |
| Post exertional malaise | 11.5 | 17.8 |  |  |  |

ID and FD symptom frequencies for 108,418 participants. % = percentage of sample endorsing the symptom. % Miss = percentage of missing data for the symptom.

* The three conditional IBS symptoms were only assessed in case IBS abdominal pain was present. Hence the numbers represent the prevalence of these symptoms in the individuals reporting abdominal pain (11.1%).

**Supplemental Figure 1.** Scree plot


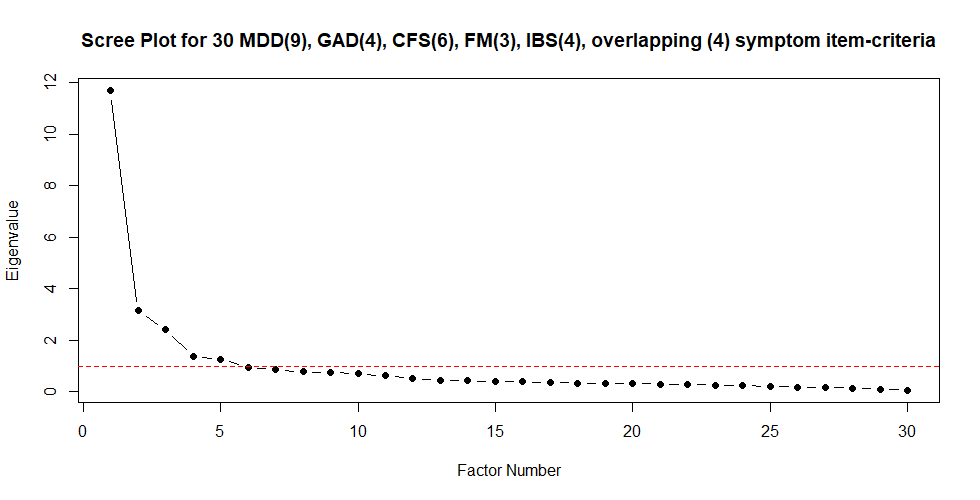


Scree plot for 30 aggregated ID and FD symptoms.

|  | F1 (IBS symptoms) | | | | F2 (Musculoskeletal pain) | | | | F3 (Depression) | | | | F4 (Anxiety) | | | | F5 (General malaise) | | | |
| --- | --- | --- | --- | --- | --- | --- | --- | --- | --- | --- | --- | --- | --- | --- | --- | --- | --- | --- | --- | --- |
| Covariate | B | β | S.E. | P-value | B | β | S.E. | P-value | B | β | S.E. | P-value | B | β | S.E. | P-value | B | β | S.E. | P-value |
| Age | 0.020 | 0.012 | 0.010 | 0.196 | **0.192** | **0.128** | **0.007** | **0.000** | **-0.157** | **-0.073** | **0.011** | **0.000** | -0.016 | -0.013 | 0.006 | 0.024 | **-0.058** | **-0.086** | **0.006** | **0.000** |
| Female gender | **-0.228** | **-0.052** | **0.009** | **0.000** | **0.327** | **0.083** | **0.006** | **0.000** | 0.103 | 0.018 | 0.009 | 0.041 | **0.291** | **0.088** | **0.005** | **0.000** | **0.141** | **0.079** | **0.005** | **0.000** |
| MDD | 0.045 | 0.004 | 0.008 | 0.667 | -0.031 | -0.003 | 0.006 | 0.667 | **5.777** | **0.347** | **0.005** | **0.000** | **1.075** | **0.112** | **0.004** | **0.000** | **0.501** | **0.097** | **0.005** | **0.000** |
| GAD | 0.051 | 0.006 | 0.008 | 0.504 | 0.167 | 0.020 | 0.007 | 0.002 | **1.970** | **0.166** | **0.006** | **0.000** | **2.916** | **0.426** | **0.004** | **0.000** | **0.610** | **0.166** | **0.005** | **0.000** |
| ME/CFS | 0.249 | 0.021 | 0.008 | 0.011 | **2.212** | **0.203** | **0.005** | **0.000** | **0.442** | **0.028** | **0.007** | **0.000** | **0.184** | **0.020** | **0.005** | **0.000** | **0.861** | **0.176** | **0.006** | **0.000** |
| FM | 0.187 | 0.022 | 0.008 | 0.008 | **2.427** | **0.314** | **0.005** | **0.000** | **0.662** | **0.059** | **0.008** | **0.000** | **0.445** | **0.069** | **0.006** | **0.000** | **0.675** | **0.194** | **0.006** | **0.000** |
| IBS | **2.395** | **0.258** | **0.006** | **0.000** | **0.583** | **0.069** | **0.005** | **0.000** | **0.530** | **0.044** | **0.007** | **0.000** | **0.329** | **0.047** | **0.005** | **0.000** | **0.638** | **0.169** | **0.007** | **0.000** |
| BMI | 0.001 | 0.000 | 0.008 | 0.991 | **0.475** | **0.101** | **0.005** | **0.000** | **0.621** | **0.091** | **0.008** | **0.000** | 0.020 | 0.005 | 0.005 | 0.289 | **0.086** | **0.041** | **0.005** | **0.000** |
| Chronic stress | **0.054** | **0.045** | **0.009** | **0.000** | **0.125** | **0.116** | **0.007** | **0.000** | **0.306** | **0.197** | **0.009** | **0.000** | **0.243** | **0.270** | **0.006** | **0.000** | **0.128** | **0.263** | **0.006** | **0.000** |
| Acute stress | -0.004 | -0.002 | 0.009 | 0.803 | **0.067** | **0.036** | **0.006** | **0.000** | **0.226** | **0.085** | **0.008** | **0.000** | **0.081** | **0.053** | **0.005** | **0.000** | **0.018** | **0.022** | **0.006** | **0.000** |
| Heavy drinking | **0.338** | **0.034** | **0.008** | **0.000** | 0.117 | 0.013 | 0.006 | 0.022 | 0.209 | 0.016 | 0.009 | 0.074 | 0.067 | 0.009 | 0.005 | 0.070 | 0.002 | 0.001 | 0.005 | 0.912 |
| CSA | 0.076 | 0.009 | 0.009 | 0.299 | 0.121 | 0.016 | 0.006 | 0.007 | **0.322** | **0.029** | **0.007** | **0.000** | **0.154** | **0.024** | **0.005** | **0.000** | **0.081** | **0.024** | **0.005** | **0.000** |
| Cancer | 0.059 | 0.006 | 0.009 | 0.457 | 0.035 | 0.004 | 0.005 | 0.446 | 0.021 | 0.002 | 0.009 | 0.849 | 0.031 | 0.004 | 0.005 | 0.385 | **0.080** | **0.021** | **0.005** | **0.000** |
| Rheumatoid arthritis | -0.055 | -0.004 | 0.008 | 0.619 | **0.798** | **0.066** | **0.005** | **0.000** | 0.390 | 0.022 | 0.008 | 0.006 | 0.016 | 0.002 | 0.005 | 0.748 | **0.097** | **0.018** | **0.005** | **0.000** |
| Osteoarthritis | **0.203** | **0.030** | **0.009** | **0.001** | **1.352** | **0.224** | **0.005** | **0.000** | 0.167 | 0.019 | 0.009 | 0.032 | **0.165** | **0.033** | **0.005** | **0.000** | **0.085** | **0.031** | **0.005** | **0.000** |
| Celiac disease | 0.667 | 0.022 | 0.007 | 0.001 | 0.144 | 0.005 | 0.006 | 0.350 | 0.510 | 0.013 | 0.008 | 0.097 | 0.088 | 0.004 | 0.005 | 0.420 | 0.078 | 0.006 | 0.005 | 0.196 |
| Ulcerative colitis | 0.557 | 0.022 | 0.008 | 0.004 | -0.109 | -0.005 | 0.005 | 0.328 | -0.235 | -0.007 | 0.008 | 0.398 | 0.048 | 0.002 | 0.005 | 0.612 | 0.104 | 0.010 | 0.005 | 0.036 |
| Heart failure | 0.021 | 0.001 | 0.008 | 0.891 | 0.278 | 0.016 | 0.005 | 0.001 | 0.385 | 0.016 | 0.009 | 0.073 | -0.007 | -0.001 | 0.005 | 0.923 | **0.215** | **0.028** | **0.005** | **0.000** |
| Crohn’s disease | **1.051** | **0.029** | **0.008** | **0.000** | 0.502 | 0.016 | 0.005 | 0.002 | 0.750 | 0.016 | 0.008 | 0.045 | -0.150 | -0.006 | 0.005 | 0.273 | 0.056 | 0.004 | 0.005 | 0.411 |
| R^2^ | 0.086 | | | | 0.428 | | | | 0.381 | | | | 0.471 | | | | 0.454 | | | |

**Supplemental Table 2.** Partial regression effect sizes of external variables

Unstandardized and Standardized partial regression effect size of covariates on the specific factors. B = unstandardized effect size; β = standardized effect size; S.E. = standard error for β. Effect for age is presented per 10 years. R^2^ = proportion of variance in the latent factor that is explained by the covariates. Estimates significant at p-value < 0.001 are bolded.


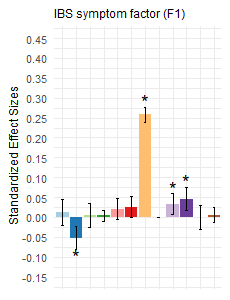


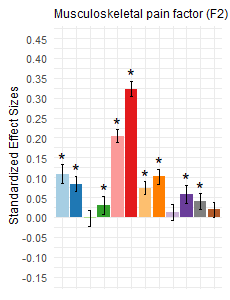

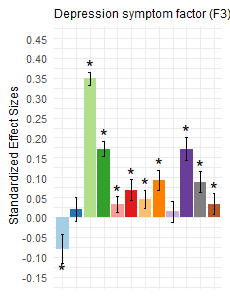

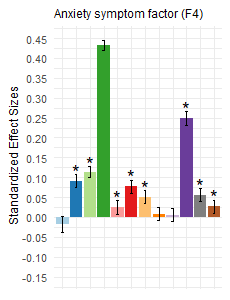

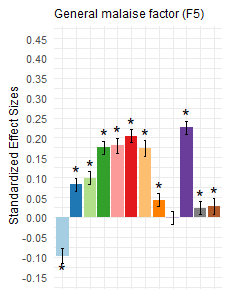

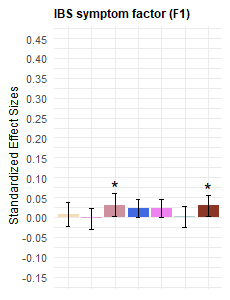

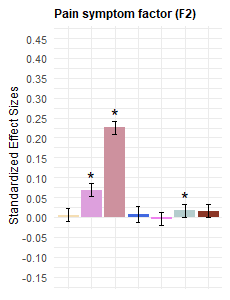

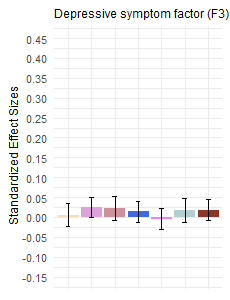

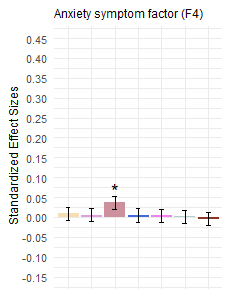

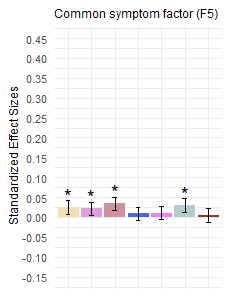

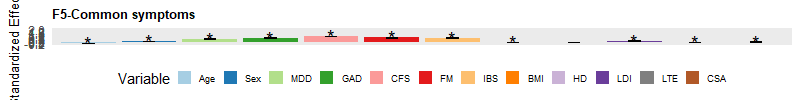

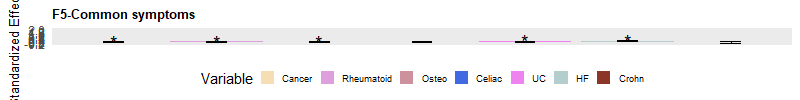


**Supplemental Figure 2**. Sensitivity analysis – Summary of CFA model standardized partial regression effect sizes

Summary of CFA model standardized partial regression effect sizes after removing illness-related stress questions from the LDI and LTE. Top row are effect sizes for risk factors and current diagnosis of IDs/FDs. Bottom row are effect sizes for self-reported presence of other medical conditions. Osteo = osteoarthritis; Celiac = Celiac disease; UC = ulcerative colitis; HF = heart failure; Crohn = Crohn’s disease. β coefficients for age are presented per 10 years. * Effect size estimates significant at the p-value < 0.001 level.

|  | F1 (IBS symptoms) | | | | F2 (Musculoskeletal pain) | | | | F3 (Depression) | | | | F4 (Anxiety) | | | | F5 (General malaise) | | | |
| --- | --- | --- | --- | --- | --- | --- | --- | --- | --- | --- | --- | --- | --- | --- | --- | --- | --- | --- | --- | --- |
| Covariate | B | β | S.E. | P-value | B | β | S.E. | P-value | B | β | S.E. | P-value | B | β | S.E. | P-value | B | β | S.E. | P-value |
| Age | 0.022 | 0.013 | 0.010 | 0.164 | **0.162** | **0.109** | **0.007** | **0.000** | **-0.173** | **-0.080** | **0.011** | **0.000** | -0.022 | -0.018 | 0.006 | 0.003 | **-0.065** | **-0.097** | **0.006** | **0.000** |
| Female gender | **-0.227** | **-0.052** | **0.009** | **0.000** | **0.332** | **0.084** | **0.006** | **0.000** | 0.113 | 0.020 | 0.009 | 0.026 | **0.299** | **0.091** | **0.005** | **0.000** | **0.145** | **0.082** | **0.005** | **0.000** |
| MDD | 0.060 | 0.005 | 0.009 | 0.584 | 0.030 | 0.003 | 0.007 | 0.695 | **5.817** | **0.349** | **0.005** | **0.000** | **1.098** | **0.114** | **0.004** | **0.000** | **0.511** | **0.099** | **0.005** | **0.000** |
| GAD | 0.033 | 0.004 | 0.009 | 0.682 | **0.249** | **0.030** | **0.006** | **0.000** | **2.037** | **0.172** | **0.006** | **0.000** | **2.955** | **0.432** | **0.004** | **0.000** | **0.641** | **0.175** | **0.005** | **0.000** |
| ME/CFS | 0.248 | 0.021 | 0.008 | 0.011 | **2.235** | **0.204** | **0.005** | **0.000** | **0.499** | **0.032** | **0.006** | **0.000** | **0.229** | **0.025** | **0.005** | **0.000** | **0.882** | **0.180** | **0.006** | **0.000** |
| FM | 0.218 | 0.026 | 0.008 | 0.002 | **2.497** | **0.323** | **0.006** | **0.000** | **0.757** | **0.068** | **0.008** | **0.000** | **0.502** | **0.077** | **0.005** | **0.000** | **0.708** | **0.204** | **0.005** | **0.000** |
| IBS | **2.401** | **0.258** | **0.006** | **0.000** | **0.614** | **0.073** | **0.005** | **0.000** | **0.559** | **0.046** | **0.007** | **0.000** | **0.364** | **0.051** | **0.005** | **0.000** | **0.654** | **0.173** | **0.006** | **0.000** |
| BMI | 0.000 | 0.000 | 0.008 | 0.994 | **0.480** | **0.102** | **0.006** | **0.000** | **0.635** | **0.093** | **0.008** | **0.000** | 0.031 | 0.008 | 0.005 | 0.097 | **0.092** | **0.043** | **0.005** | **0.000** |
| Chronic stress | **0.060** | **0.046** | **0.009** | **0.000** | **0.068** | **0.058** | **0.007** | **0.000** | **0.292** | **0.171** | **0.009** | **0.000** | **0.244** | **0.249** | **0.005** | **0.000** | **0.118** | **0.226** | **0.005** | **0.000** |
| Acute stress | -0.001 | 0.000 | 0.009 | 0.978 | **0.076** | **0.040** | **0.006** | **0.000** | **0.243** | **0.089** | **0.008** | **0.000** | **0.089** | **0.056** | **0.005** | **0.000** | **0.019** | **0.023** | **0.005** | **0.000** |
| Heavy drinking | **0.334** | **0.033** | **0.008** | **0.000** | 0.108 | 0.012 | 0.006 | 0.036 | 0.181 | 0.014 | 0.008 | 0.103 | 0.049 | 0.006 | 0.005 | 0.229 | -0.007 | -0.002 | 0.005 | 0.728 |
| CSA | 0.049 | 0.006 | 0.009 | 0.531 | 0.145 | 0.019 | 0.006 | 0.001 | **0.361** | **0.033** | **0.008** | **0.000** | **0.169** | **0.027** | **0.005** | **0.000** | **0.091** | **0.027** | **0.006** | **0.000** |
| Cancer | 0.064 | 0.007 | 0.009 | 0.421 | 0.050 | 0.006 | 0.005 | 0.269 | 0.079 | 0.006 | 0.009 | 0.484 | 0.065 | 0.009 | 0.005 | 0.072 | **0.095** | **0.025** | **0.005** | **0.000** |
| Rheumatoid arthritis | -0.047 | -0.003 | 0.008 | 0.672 | **0.819** | **0.068** | **0.005** | **0.000** | 0.446 | 0.025 | 0.008 | 0.001 | 0.056 | 0.006 | 0.005 | 0.263 | **0.119** | **0.022** | **0.005** | **0.000** |
| Osteoarthritis | 0.203 | 0.031 | 0.009 | 0.001 | **1.360** | **0.226** | **0.005** | **0.000** | 0.193 | 0.022 | 0.009 | 0.013 | **0.186** | **0.037** | **0.005** | **0.000** | **0.094** | **0.035** | **0.005** | **0.000** |
| Celiac disease | 0.678 | 0.022 | 0.007 | 0.001 | 0.179 | 0.007 | 0.006 | 0.241 | 0.541 | 0.014 | 0.008 | 0.074 | 0.117 | 0.005 | 0.005 | 0.291 | 0.106 | 0.009 | 0.005 | 0.105 |
| Ulcerative colitis | 0.557 | 0.022 | 0.007 | 0.003 | -0.090 | -0.004 | 0.005 | 0.453 | -0.184 | -0.005 | 0.008 | 0.517 | 0.074 | 0.004 | 0.005 | 0.431 | 0.118 | 0.011 | 0.005 | 0.019 |
| Heart failure | 0.030 | 0.002 | 0.008 | 0.850 | **0.295** | **0.017** | **0.005** | **0.001** | 0.439 | 0.018 | 0.009 | 0.043 | 0.033 | 0.002 | 0.005 | 0.669 | **0.231** | **0.030** | **0.005** | **0.000** |
| Crohn’s disease | **1.066** | **0.030** | **0.008** | **0.000** | 0.510 | 0.016 | 0.005 | 0.002 | 0.825 | 0.018 | 0.008 | 0.026 | -0.098 | -0.004 | 0.005 | 0.475 | 0.077 | 0.005 | 0.005 | 0.279 |
| R^2^ | 0.085 | | | | 0.417 | | | | 0.374 | | | | 0.463 | | | | 0.437 | | | |

**Supplemental Table 3.** Sensitivity analysis -- partial regression effect sizes of external variables

Unstandardized and Standardized partial regression effect size of covariates on the specific factors after removing illness-related stress questions from the LDI and LTE. B = unstandardized effect size; β = standardized effect size; S.E. = standard error for β. Effect for age is presented per 10 years. R^2^ = proportion of variance in the latent factor that is explained by the covariates. Estimates significant at p-value < 0.001 are bolded.

**References**

Brugha, T. S., & Cragg, D. (1990). The List of Threatening Experiences: the reliability and validity of a brief life events questionnaire. *Acta psychiatrica Scandinavica*, *82*(1), 77–81. https://doi.org/10.1111/j.1600-0447.1990.tb01360.x

Rosmalen, J. G., Bos, E. H., & de Jonge, P. (2012). Validation of the Long-term Difficulties Inventory (LDI) and the List of Threatening Experiences (LTE) as measures of stress in epidemiological population-based cohort studies. *Psychological medicine*, *42*(12), 2599–2608. https://doi.org/10.1017/S0033291712000608

Saini, U., Rosmalen, J. G. M., Oldehinkel, A. J., & van Loo, H. M. (2024). Connecting the dots: Network structures of internalizing and functional symptoms in a population-based cohort. *Journal of psychosomatic research*, *187*, 111932.

Statistics Netherlands, National Institute for Public Health and the Environment, & Trimbos Institute (2024). https://www.rivm.nl/leefstijlmonitor/alcoholgebruik-onder-volwassenen

Thombs, B. D., Bernstein, D. P., Lobbestael, J., & Arntz, A. (2009). A validation study of the Dutch Childhood Trauma Questionnaire-Short Form: factor structure, reliability, and known-groups validity. *Child abuse & neglect*, *33*(8), 518–523. https://doi.org/10.1016/j.chiabu.2009.03.001
